# Supplementary material for: MpoxRadar: a worldwide MPXV genomic surveillance dashboard
Source: Nucleic Acids Res. 2023 May 11;51(W1):W331–7. doi: 10.1093/nar/gkad325 (PMC10320163; doi:10.1093/nar/gkad325)
Supplement: gkad325_Supplemental_File [file gkad325_supplemental_file.pdf]

OXFORD ACADEMIC | Journals

Supplementary Information for

MpoxRadar: a worldwide Mpox genomic surveillance dashboard

Ferdous Nasri, Kunaphas Kongkitimanon [‡], Alice Wittig [‡], Jorge Sánchez Cortés, Annika Brinkmann, Andreas Nitsche, Anna-Juliane Schmachtenberg [†], Bernhard Y. Renard [†], Stephan Fuchs [†]

Stephan Fuchs.
E-mail: FuchsS@rki.de

This PDF file includes:

Tables S1 to S2

Table S1. MpoxSonar commands available via the web application.

Name	Description
`list-prop`	View all properties added to the database.
`match`	Query sequences based on profiles or properties. The match command alone gets all mutation profiles from the database.

Table S2. Examples of queries enabled by the MpoxSonar command options.

Description	Command
Query all samples aligned to reference genome “NC_063383.1” from USA	`match -r NC_063383.1 --COUNTRY USA`
Query all samples with nucleotide deletion mutations in the first 60 bp (e.g., del:1-60, del:1-6, del:11-20)	`match --profile del:1-60`
Query all samples except samples that contain ”C162331T” nucleotide mutation	`match --profile ^C162331T`
Query all samples with an amino acid mutation ”L246F” with alignments to all references. Note, multiple commands are passed with the logical `OR` when the `-profile` tag is used separately.	`match --profile OPG188:L246F -profile MPXV-UK_P2-164:L246F --profile MPXV-USA_2022_MA001-164:L246F`
Query all samples with a nucleotide mutation ”A151461C” and a deletion mutation on (exactly) the first 6 bp. Note, multiple commands passed with a singular `-profile` tag are processed with the logical `AND`.	`match -profile A151461C del:=1-=6`
Query all samples with a sequence length in a range between 19710 and 19720 bp	`match --LENGTH >19710 <19720`
Query a sample directly using the sample ID	`match --sample ON585033.1`
